# An Ultrastructural, In-Situ Study on the Impact of Desensitizing Agents on Dentin

**DOI:** 10.1016/j.identj.2024.09.027

**Published:** 2024-10-09

**Authors:** Anton Schestakow, Gerrit Josef Lefering, Matthias Hannig

**Affiliations:** Clinic of Operative Dentistry, Periodontology and Preventive Dentistry, Saarland University, Homburg/Saar, Germany

**Keywords:** Dental pellicle, Dentin desensitizing agents, Electron microscopy, Ultrastructure

## Abstract

**Introduction and aims:**

With the increasing prevalence of dentin hypersensitivity, more and more desensitizing agents with tubule-occluding properties are advocated in the market. The aim of the present study was to investigate the deposition of these agents on the dentin surface under in-situ conditions.

**Methods:**

Bovine dentin specimens were pretreated with phosphoric acid and fixed to individual upper splints that were carried by up to 2 subjects for 3 min to allow pellicle formation. The desensitizing agents containing either calcium carbonate and arginine, casein-phosphopeptide amorphous calcium phosphate, zinc-carbonate hydroxyapatite, tetracalcium phosphate and dicalcium phosphate anhydrous or hydroxyapatite nanoparticles were applied ex situ. Specimens without treatment served as controls. After a further 6 h of intraoral exposure, specimens were removed and analysed by scanning (n = 4 specimens per substance, in total) and transmission electron microscopy (n = 2 specimens per substance).

**Results:**

Application of desensitizing agents resulted in the deposition of different structures on the dentin surface and occlusion of dentinal tubules.

**Conclusion:**

The ultrastructural analysis using transmission electron microscopy indicates that dentinal tubules were occluded under in-situ conditions not only by inorganic but also by organic deposits from the oral cavity.

## Introduction

With the increasing prevalence of dentin hypersensitivity, more and more therapeutic agents are advocated in the market. Dentin hypersensitivity is the result of dentin exposure to the oral cavity that occurs mainly in the cervical area of teeth. When dentin is exposed after loss of enamel or after gingival recession and subsequent loss of cementum, the dentin becomes sensitive to external stimuli.^1^ According to the hydrodynamic theory, external stimuli lead to fluid movement in the dentinal tubules that is processed by odontoblasts and nerve fibres into pain sensations.^2^^,^^3^ The reduction of neuronal transmission and the occlusion of permeable dentinal tubules are 2 noninvasive treatment concepts. Since the last century, potassium salts have been investigated as desensitisers that prevent the conduction of action potentials by depolarisation of nerve terminals in the pulp. However, the effect is short, as the potassium ions are rapidly removed by the physiological outward flow in the dentinal tubules.^4^ Since fluid movement is facilitated when dentinal tubules are open, dentin hypersensitivity can be also treated by occlusion of open tubules. There are many desensitizing agents with tubule-occluding properties whose long-term effects were confirmed in clinical studies, but a gold standard is not yet determined.^5^ These agents have the property of interacting with the dental hard tissue, leading to deposition and occlusion of open tubules. While the reduction of dentin hypersensitivity is tested by thermal, tactile or the most reproducible evaporative stimuli, in which a stimulus is generated by an air spray,^5^ the degree of tubule occlusion can be examined by measurements of permeability,^6^ or by microscopy and other imaging procedures. Therefore, dentin specimens of human or bovine origin are treated with desensitizing agents and then prepared for the analysis. The occlusion of tubule orifices at the dental surface and the depth of penetration into the dentinal tubules can be examined using scanning electron microscopy (SEM). Confocal laser scanning microscopy (CLSM) is another method to assess the permeability of dentinal tubules, which is associated with the degree of tubule occlusion.^7^ In contrast to SEM and CLSM, which generate 2-dimensional images, high-resolution X-ray computed tomography (nano-CT) or focused ion beam-scanning electron microscopy (FIB-SEM) can be used for 3-dimensional reconstruction of the entire sample.^8^^,^^9^ Although the effectiveness of many desensitizing agents with tubule-occluding properties was already confirmed by clinical studies,^5^ the resolution of the above-mentioned microscopes is not sufficient to investigate the interaction of the desensitizing agents with the dental surface in detail. In addition, dental surfaces are covered by a 10 to 1000 nm thick layer of adsorbed salivary proteins, the pellicle, which can modify the interaction of substances with the dental surface.^10^, ^11^, ^12^ Previous work has focused on the degree of occlusion and penetration of dentinal tubules by desensitizing agents. There is a plethora of agents with tubule-occluding properties such as strontium salts, fluorides, oxalates, and calcium phosphate compounds.^3^ Calcium phosphate compounds are of great interest in dental research thanks to their biocompatibility and remineralizing properties.^13^^,^^14^ One of the main issues in our knowledge of these substances is their specific interaction with the dentin surface under oral conditions. Consequently, the present study aimed to extend current knowledge of desensitizing agents with tubule-occluding properties by applying transmission electron microscopy (TEM), which outperforms previous microscopic analyses in terms of resolution, and by employing an in-situ study design taking the salivary pellicle into account. The aim of the present study was to investigate the interaction of common tubule-occluding desensitizing agents with the dentin surface under in-situ conditions after a single application to pellicle-covered dentin and subsequent intraoral exposure.

## Material and methods

### Specimen preparation

Lower incisor teeth were extracted from 2-year-old calves from a local slaughterhouse (Schlachthaus, Zweibrücken, Germany). The roots were removed using diamond cutting discs (Schott Diamant, Oldendorf, Germany), the crowns were cut in half along the frontal plane, and the labial fragments were then processed into dentin samples using a saw (Conrad Apparatebau Clausthal, Clausthal-Zellerfeld, Germany). The samples were processed to specimens (3.5 mm × 6 mm) and polished to 2500 grit with a wet grinding machine (Buehler, Duesseldorf, Germany) and silicon carbide grinding papers (Buehler, Duesseldorf, Germany). The smear layer was removed by 3% NaOCl (Hedinger, Stuttgart, Germany) for 30 seconds and ultrasonication in sterile water for 2 minutes. Disinfection was performed in 70% isopropyl alcohol (Hedinger, Stuttgart, Germany) for 15 minutes and rehydration in sterile water (B. Braun, Melsungen, Germany) for 6 hours. After application of a 37.5% phosphoric acid gel (Kerr, Scafati, Italy) to the dentin surface for 30 seconds and rinsing with sterile water to open the dentinal tubules, specimens were fixed with silicone impression material (President light body, Coltene/Whaledent, Langenau, Germany) on individual upper splints made of methacrylate (Scheu Dental, Iserlohn, Germany). Specimens for each treatment group were taken from a pool of prepared samples.

### Subjects

Two members of our laboratory staff (24 and 30 years, male and female, respectively) participated in the study by wearing individual splints with dentin specimens. A medical history and examination were performed to rule out systemic diseases, carious lesions, periodontitis, diseases of the oral mucosa or salivary glands, and any medication intake. Informed written consent was obtained from both participants. The study was performed in accordance with the Declaration of Helsinki, and was approved by the ethic committee of the Medical Association of Saarland (238/03, 2016).

### In-situ trial

**Fig. 1:**
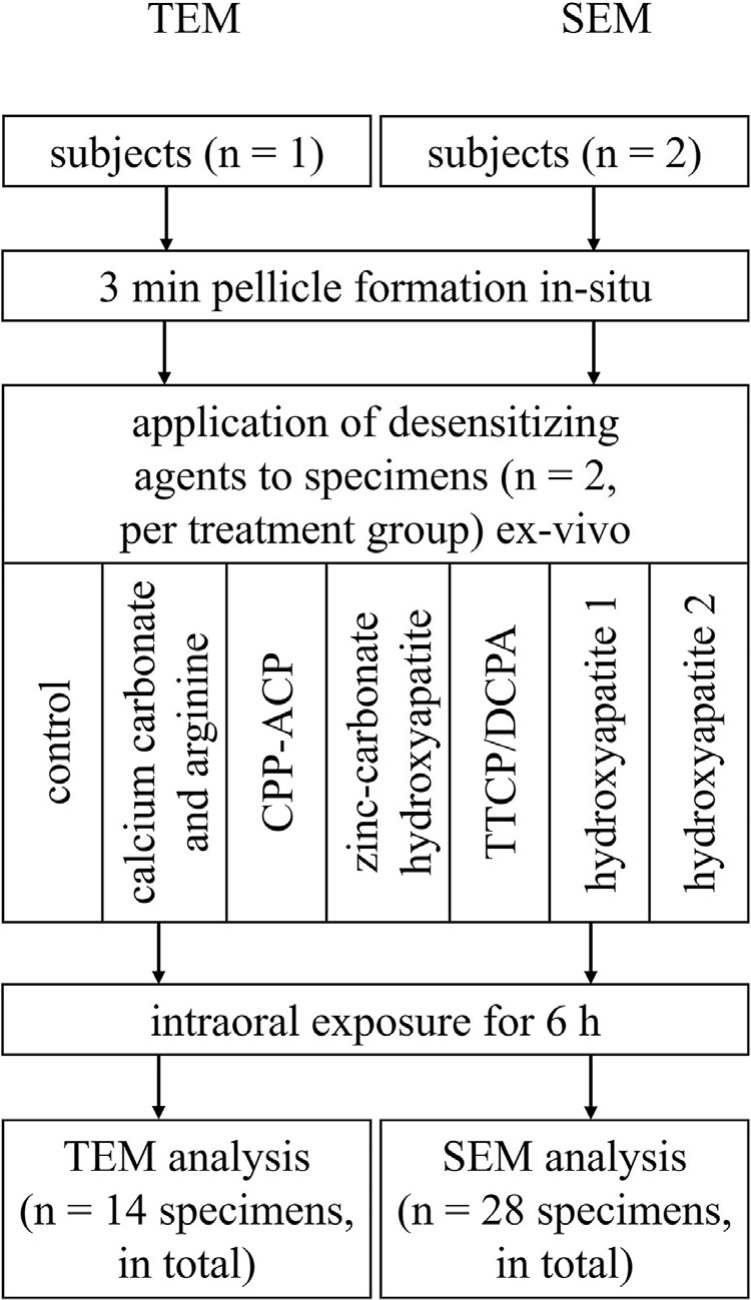
Flow chart of the in-situ trial.

For each treatment group, 2 specimens were attached to the splints (Figure 1). With one subject for TEM and 2 subjects for SEM, a total of 14 and 28 specimens were evaluated, respectively. The splints were inserted into the oral cavity at 8 AM for 3 minutes to allow initial pellicle formation, before test substances were applied with a microbrush (Microbrush International, Heidelberg, Germany), ex-vivo. The test substances included products with the active ingredients calcium carbonate and arginine; casein-phosphopeptide amorphous calcium phosphate (CPP-ACP); zinc-carbonate hydroxyapatite; tetracalcium phosphate and dicalcium phosphate anhydrous (TTCP/DCPA); and 2 types of hydroxyapatite nanoparticles produced using the same method but by different manufacturers (Table 1). After specimens were gently rinsed with sterile water, splints were worn intraorally for a further 6 h. In the control, specimens were not treated with a test substance. During the in-situ trial, subjects were allowed to drink water and store the splints in a humid chamber for eating. Finally, specimens were removed from the oral cavity, rinsed with sterile water and prepared for the respective analysis.

**Table 1:** Test substances.

Active substance	Product	All ingredients	Manufacturer	Application
calcium carbonate, arginine	Elmex Sensitive Professional (dentifrice)	calcium carbonate, aqua, sorbitol, arginine, sodium lauryl sulfate, aroma, sodium monofluorophosphate, cellulose gum, sodium bicarbonate, tetrasodium pyrophosphate, sodium saccharin, benzyl alcohol, xanthan gum, limonene, CI 77891	CP GABA, Hamburg, Germany	Apply for 2 min
casein-phosphopeptide amorphous calcium phosphate (CPP-ACP)	GC MI Paste Plus (dentifrice)	pure water, glycerol, casein-phosphopeptide amorphous calcium phosphate, D-sorbitol, CMC-Na, propylene glycol, silicon dioxide, titanium dioxide, xylitol, phosphoric acid, sodium fluoride, flavouring, sodium saccharin, ethyl p-hydroxybenzoate, propyl p-hydroxybenzoate, butyl p-hydroxybenzoate	GC Europe, Leuven, Belgium	Apply for 2 min
zinc-carbonate-hydroxyapatite	Biorepair Plus (dentifrice)	aqua, zinc-carbonate hydroxyapatite, glycerin, sorbitol, hydrated silica, silica, cocamidopropyl betaine, cellulose gum, aroma, lactoferrin, sodium myristoyl sarcosinate, sodium methyl cocoyl taurate, hamamelis virginiana leaf extract, spirulina platensis extract, calendula officinalis flower extract, zinc PCA, sodium hyaluronate, tocopheryl acetate, retinyl palmitate, sodium saccharin, phenoxyethanol, benzyl alcohol, sodium benzoate, potassium sorbate, limonene, CI 77891	Dr. Kurt Wolf, Bielefeld, Germany	Apply for 2 min
tetracalcium phosphate, dicalcium phosphate anhydrous (TTCP/DCPA)	Teethmate Desensitiser (powder and liquid)	powder (tetracalcium phosphate, dicalcium phosphate anhydrous), liquid (water, preservatives)	Kuraray Europe, Hattersheim am Main, Germany	Mix powder and liquid for hydroxyapatite formation; apply and wait 2 min
hydroxyapatite 1	Hydroxyapatite	hydroxyapatite nanoparticles (median size 100 nm, needle-shaped)	Kalident, Kalichem Italia, Rezzato, Italy	Mix powder with water (1:1); apply for 2 min
hydroxyapatite 2	Hydroxyapatite	hydroxyapatite nanoparticles (size 100-300 nm)	Chemische Fabrik Budenheim, Budenheim, Germany	Mix powder with water (1:1); apply for 2 min

The product compositions were obtained from the packaging information.

### TEM

Specimens were fixed in a fixing solution containing 1% glutaraldehyde (Serva Electrophoresis), 1% paraformaldehyde (Science Services) and 0.1 M cacodylate (Carl Roth) for 1 hour, post-fixed in 2% osmium tetroxide (Electron Microscopy Sciences) for 2 hours and dehydrated in an ascending alcohol series (Fisher Scientific). Then, specimens were embedded in Araldite CY 212 (Serva) and 50 nm thin sections were cut using an ultramicrotome (Reichert) with a diamond knife (Diatom 45). The sections were contrasted with uranyl acetate (Ted Pella) and lead citrate (Merk), and examined using TEM (TEM Tecnai 12 BioTwin, FEI Company) at up to 49,000-fold magnification regarding the interaction of the test substance with the dentin surface, with 9 to 42 images taken per specimen.

### SEM

Specimens were air-dried overnight, sputter-coated with carbon (Micro to Nano, Haarlem, The Netherlands), and examined for the occlusion of dentinal tubules using SEM (XL 30 ESEM FEG, FEI Company, Eindhoven, The Netherlands) at up to 20,000-fold magnification, with 6 images taken of one random spot per specimens.

## Results

### TEM

**Fig. 2:**
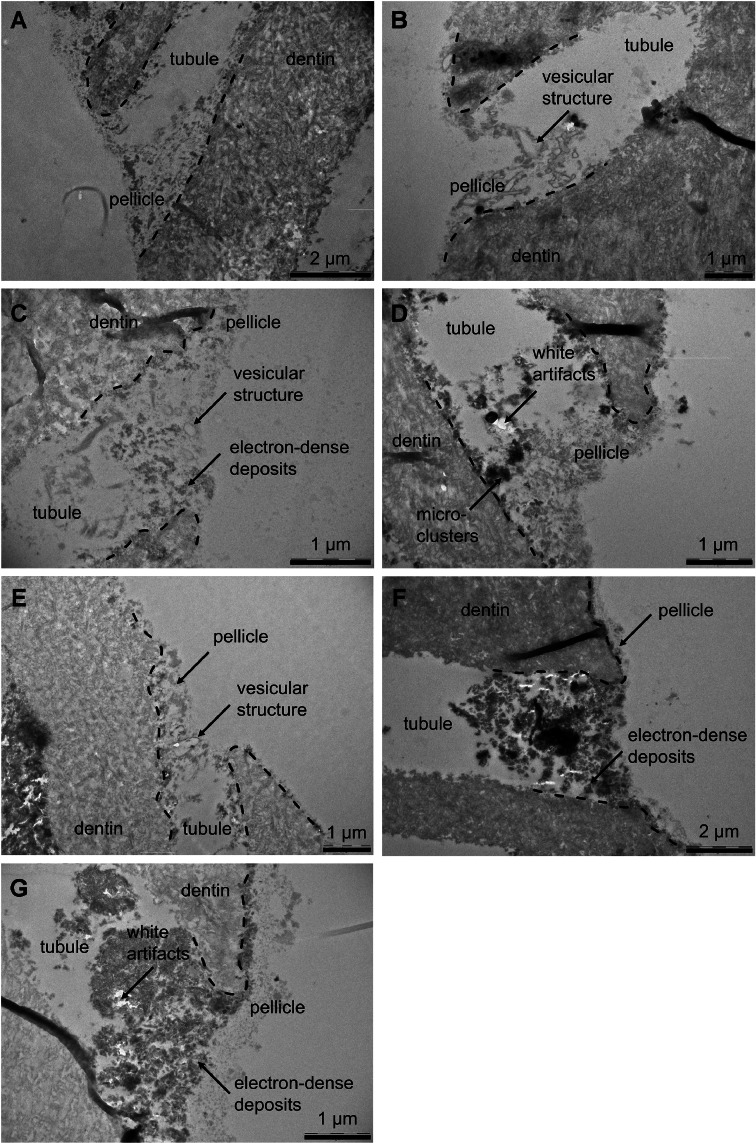
Transmission electron micrographs of the intratubular dentin of specimens after pellicle formation for 3 minutes, application of desensitizing agents, and an additional 6 hours of intraoral exposure. The dentin was covered by a loosely structured pellicle. Note the vesicular structures, electron-dense deposits, and white artifacts after application of test substances. (A) control; (B) calcium carbonate, arginine; (C) casein-phosphopeptide amorphous calcium phosphate; (D) zinc-carbonate-hydroxyapatite; (E) tetracalcium phosphate, dicalcium phosphate anhydrous; (F) hydroxyapatite 1; (G) hydroxyapatite 2.

**Fig. 3:**
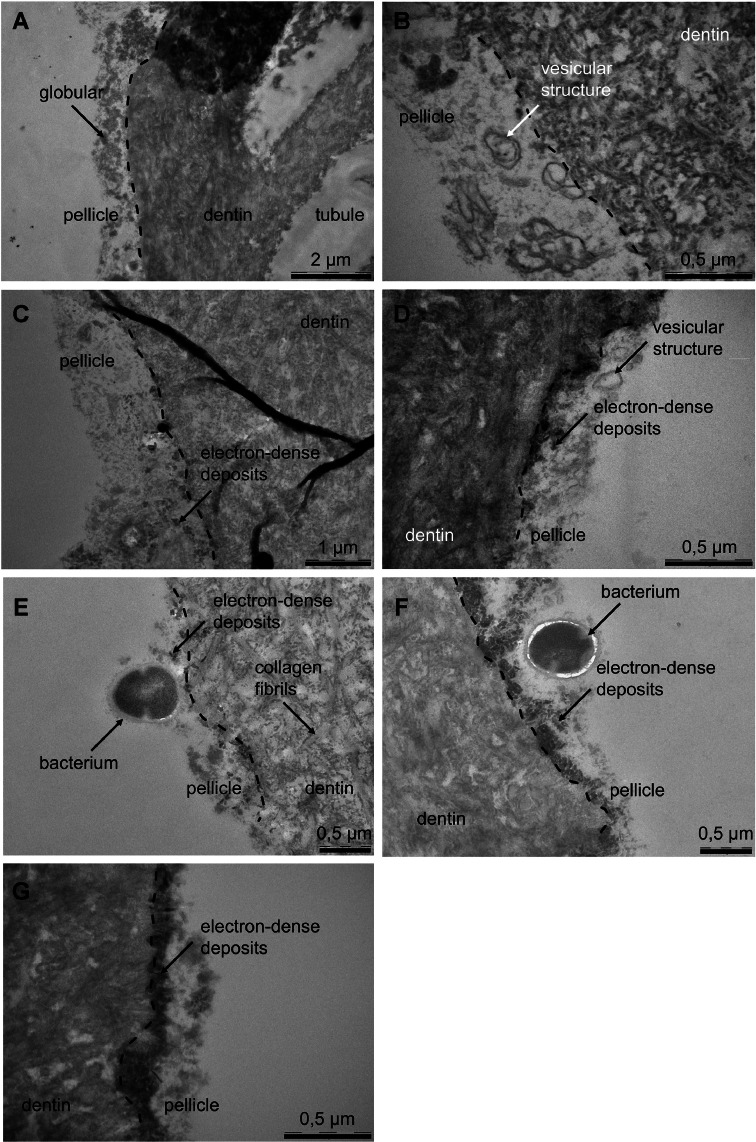
Transmission electron micrographs of the intertubular dentin of specimens after pellicle formation for 3 minutes, application of desensitizing agents, and an additional 6 hours of intraoral exposure. The dentin was covered by a loosely structured pellicle. Note the vesicular structures and electron-dense deposits after application of test substances. (A) control; (B) calcium carbonate, arginine; (C) casein-phosphopeptide amorphous calcium phosphate; (D) zinc-carbonate-hydroxyapatite; (E) tetracalcium phosphate, dicalcium phosphate anhydrous; (F) hydroxyapatite 1; (G) hydroxyapatite 2.

The effect of desensitizing agents on the pellicle covered intratubular and intertubular dentin is shown in Fig. 2, Fig. 3, respectively. After dentin specimens were pretreated with phosphoric acid, the dentinal tubules were opened and the dentin surface was demineralised. The demineralised dentin is marked by exposed collagen fibrils, which appear needle-shaped when cut lengthwise and round when cut crosswise. These organic fibrils are less electron-rich compared to the inorganic matrix. The thin organic pellicle layer was able to form on the intertubular dentin and particularly in the tubule orifices during a 6-hours intraoral exposure. This pellicle is composed of globular protein aggregates and a loose, granular protein network, resulting in a typically heterogeneous structure. The pellicle infiltrated the dentinal tubules by a few µm. Furthermore, a few bacteria were observed after 6 h, which adhered to the pellicle through their fimbriae (Figures 2A and3A). The morphology of the pellicle was modified when dentin specimens were treated with calcium carbonate and arginine after initial pellicle formation. Vesicular structures with an electron-deficient core and an electron-dense coat with attached electron-dense particles appeared in the pellicle. Both, the vesicles and the pellicle occluded the tubule orifices (Figures 2B and3B). A similar observation was made after the application of CPP-ACP, but in addition angular and electron-dense particles appeared. They were aggregated and distributed throughout the diameter of the pellicle, and formed an electron-dense layer on the demineralised dentin (Figures 2C and3C). Treatment with zinc-carbonate-hydroxyapatite also resulted in deposition of particles with an electron-dense layer on the dentin. Occasionally, white artifacts appeared next to the electron-dense structures, representing large particles that were lost during ultrathin sectioning (Figures 2D and3D). When dentin specimens were treated with TTCP/DCPA, both crystalline and vesicular structures occluded the tubule orifices. In contrast, the vesicular structures were not round-shaped but elongated. The particles were distributed throughout the pellicle that was formed on the intertubular dentin (Figures 2E and3E). Hydroxyapatite 1 and 2 resulted in complete occlusion of tubule orifices through substantial deposition of electron-dense particles. The particles were found throughout the pellicle but were predominantly deposited on the dentin surface, forming a dense layer of agglutinated particles. Pellicle formation was not affected by the application of hydroxyapatite 1 and 2 (Figures 2F and G; Figures 3F and G). Regardless of the treatment, the pellicle mostly had a granular and globular morphology and was partially modified by incorporation of different structures, with the electron-dense particles enriched in the basal layers of the pellicle and the vesicular structures in the superficial pellicle layer.

### SEM

**Fig. 4:**
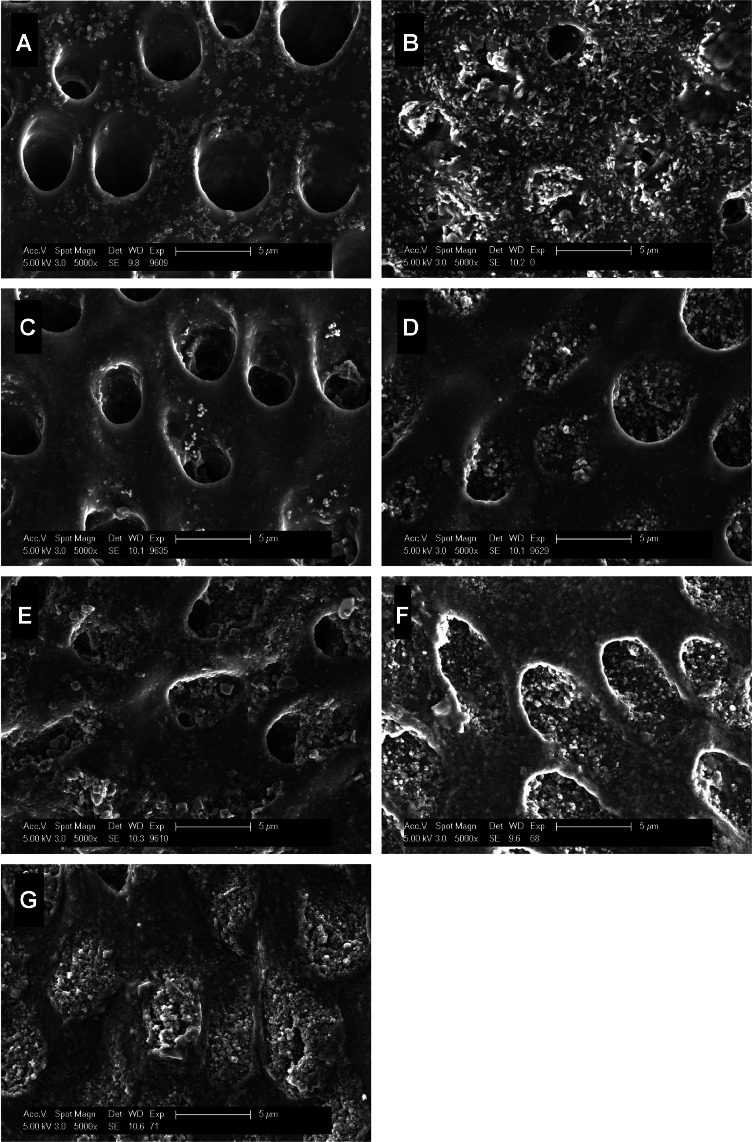
Scanning electron micrographs of dentin-specimens after pellicle formation for 3 minutes, application of test substances, and an additional 6 hours of intraoral exposure. (A) control; (B) calcium carbonate, arginine; (C) casein-phosphopeptide amorphous calcium phosphate; (D) zinc-carbonate-hydroxyapatite; (E) tetracalcium phosphate, dicalcium phosphate anhydrous; (F) hydroxyapatite 1; (G) hydroxyapatite 2. Note the open (A, C), partially (B, D, E) or completely occluded (F, G) dentinal tubules.

When dentin specimens were pretreated with phosphoric acid, the dentinal tubules were completely opened and remained open after 6 h of intraoral exposure (Figure 4). A similar morphology was observed after the application of CPP-ACP, with only a few aggregates deposited in the dentinal tubules. Calcium carbonate and arginine, zinc-carbonate-hydroxyapatite, and TTCP/DCPA resulted in deposition of many intratubular aggregates, with calcium carbonate and arginine leading to additional intertubular deposits. Some dentinal tubules were completely occluded, and even more so after using hydroxyapatite 1 or 2. Irrespective of the desensitizing agents used, the deposits consisted primarily of agglomerates rather than distinct crystallites or particles.

## Discussion

Treatment of dentin specimens with desensitising agents in-situ led to deposition of vesicular and crystalline structures on the dentin surface and in the pellicle even after 6 hours of intraoral exposure, contributing to the occlusion of tubule orifices in comparison to the control. The present study design improves previous methods by taking the pellicle into account, which is able to form under oral conditions. Furthermore, TEM has a high resolution for visualisation of the deposited organic and inorganic components on the dentin surface and especially in the pellicle. In contrast to mouthwashes, ex-vivo application of dentifrices or thick suspensions is similar to in-vivo application with cotton rolls isolation and ensure standardised treatment.

Calcium carbonate and arginine are effective in reducing dentin hypersensitivity thanks to a bio-inspired approach. Saliva contains calcium, phosphate, and glycoproteins that can occlude dentinal tubules by forming precipitating aggregates. However, this process is slow and susceptible to acids.^15^ Based on the natural mechanism, the complex of calcium carbonate and arginine was developed. The positively charged arginine adheres to the negatively charged dental surface and facilitate the deposition of calcium carbonate, which is supported by the high pH value of the complex. In contrast to salivary deposits, the occlusion with calcium carbonate and arginine is also acid-resistant.^16^^,^^17^ In-vitro studies and the present in-situ study indicate that the desensitizing property is based on the occlusion of tubule orifices,^18^, ^19^, ^20^ however, it is not clear to what extent calcium carbonate is involved, especially since hardly any electron-dense structures representing mineral deposits were observed by TEM. The vesicular structures integrated in the pellicle indicate that Elmex Sensitive Professional lead to an organic rather than an inorganic occlusion of dentinal tubules, which might be responsible for the instant hypersensitivity relief observed in clinical studies.^21^ The vesicular structures can be agglomerates of calcium carbonate and arginine first proposed by Kleinberg,^16^ lipids in the pellicle,^22^ or the surfactant sodium dodecyl sulphate that is contained in dentifrices such as Elmex Sensitive Professional and able to form micelles in aqueous solutions.^23^ In a study by Lavender et al.^20^ dentin specimens were treated with a similar dentifrice in-vitro and analysed using confocal laser scanning microscopy and electron spectroscopy. The amino acid arginine stained with a fluorescence probe accumulated in the tubule orifices and, as chemical analysis showed, also facilitated the incorporation of calcium carbonate. The extent to which calcium carbonate plays a role in the long-term effects observed in clinical studies merit further ultrastructural investigation.

Treatment of specimens with CPP-ACP resulted in deposition of few electron-dense structures and vesicles on the dentin and in the pellicle, representing precipitated calcium phosphate and casein micelles, respectively.^24^ CPP is able both to stabilise clusters of ACP in a supersaturated state and to adhere to the dental surface and particularly the organic matrix of dentin, resulting in enrichment and precipitation of calcium and phosphate at the dental surface.^25^^,^^26^ The deposits were not only observed in the basal layer of the pellicle, but were distributed throughout the pellicle layer, suggesting that they may were loosely attached to the 3-min pellicle, detached during further intraoral exposure, and again participated in pellicle formation. Similar to ultrastructural analysis of CPP-ACP treated enamel pellicles, the electron-dense particles were homogenously arranged and had a high affinity to the pellicle.^27^ However, the tubule orifices remained mostly open. According to in-vitro studies, the tubule-occluding properties of a CPP-ACP containing dentifrice depend on the frequency of application and the presence of fluoride, with the more frequent application of a fluoride-free dentifrice leading to further occlusion of tubule orifices.^28^, ^29^, ^30^, ^31^ GC MI Pate Plus of the present study contains fluoride, which can inactivate the complex by interaction with ACP.^32^

The application of zinc-carbonate-hydroxyapatite led to the formation of an electron-dense layer on the dentin and to partial occlusion of the tubule orifices. Incorporation of synthetic hydroxyapatite into dentifrices is considered a biomimetic approach, with additional zinc acting as a bifunctional ion that both provides antibacterial effects and promote adhesion to the dental hard tissue,^33^ but the latter may be affected by the presence of the salivary pellicle. The electron-dense particles were not evenly distributed but formed microclusters as described in the literature.^27^^,^^34^ Agglutination is a common issue in nanoparticles due to the high surface energy, which is supported by the dipole character of hydroxyapatite.^35^^,^^36^ Although the desensitizing effect of zinc-carbonate-hydroxyapatite was demonstrated in a clinical trial,^37^ neither the present nor in-vitro studies from the literature showed a complete occlusion of tubule orifices.^19^^,^^38^^,^^39^ In the in-situ study by Alsherbiney et al.,^40^ however, almost half of all tubules were completely occluded after brushing with a similar dentifrice twice a day for 1 d and up to 8 weeks. But when evaluating the representative micrographs of the study by Alsherbiney et al.,^40^ it appeared that although the tubular diameter decreased after the treatment, the tubule orifices remained open, indicating that even a partial occlusion reduces dentin hypersensitivity.

While the other desensitizing agents induce precipitation of calcium and phosphates or deposition of precipitates, TTCP/DCPA is a different approach. As already postulated in the patent by Brown et Chow,^41^ mixing TTCP and DCPA in a phosphate-containing solution result in the formation of biomimetic hydroxyapatite. During the preparation, TTCP dissolves in the solution and later reprecipitates onto existing crystals such as DCPA, forming needle-shaped hydroxyapatite.^42^ In the in-vitro study by Zhou et al.,^43^ the dentinal tubules were clearly filled or even completely occluded after the application of TTCP/DCPA. In the present in-situ study, however, only a few needle-shaped particles were observed, which may be attributed to the pellicle formed prior to the application. The extent to which natural built-in hydroxyapatite can grow under these conditions merit further investigation.

The experimental approaches with hydroxyapatite nanoparticles completely occluded tubule orifices. The high affinity of specific salivary proteins and the small particle size support the adhesion of hydroxyapatite nanoparticles to the pellicle.^11^^,^^44^ In the presence of saliva, hydroxyapatite nanoparticles agglomerate into clusters that can also contribute to occlusion of tubule orifices.^45^ Different in-vitro and one in-situ study demonstrated the occlusion with nanosised hydroxyapatite using SEM,^7^^,^^46^, ^47^, ^48^ which was verified by the present study. In addition, the high resolution of TEM and the use of ultrathin sections allowed the analysis of the subsurface occlusion and interaction with the pellicle and dental hard tissue. Application of hydroxyapatite nanoparticles led to substantial deposition of densely packed particles in the basal pellicle layer and dentinal tubules, but it should be noted that both experimental solutions with 50% hydroxyapatite contain distinctly more active ingredients than the commercial desensitizing agents. An infiltration of the demineralised dentin, however, was not observed. Even though ultrastructural analyses suggest semi-permeable properties of the pellicle,^49^ the permeability of the pellicle was not tested yet, and it remains unclear to what extent the pellicle is permeable to high-molecular-weight or solid substances.

The qualitative results of the present exploratory study should be considered in light of some limitations. The main limitation is the low number of subjects and specimens, which limits generalisation of the results but provides first insights into the interaction of common desensitizing agents with the dentin surface in situ. The substances were only applied once, although clinical studies suggest that the efficacy increases after repeated application, for example, the use of arginine does not have an immediate but a medium and long-term effect.^5^ Another limitation is the use of a microbrush for application without rinsing, which partly differs from the manufacturer's instructions. The extent to which the mode of application influences the efficacy of the desensitizing agents is unclear and requires further investigation. Furthermore, dentin specimens were of bovine origin and even though the target tissue is human dentin, bovine teeth have fewer variations as they are extracted at the same age as calves, which are also fed the same diet.^50^ The final limitation of the present study is the use of desensitizing substances with different compositions and concentrations of the active ingredients. Therefore, it was not possible to compare the substances with each other. The in-situ trial was conducted several years ago, and the desensitizing agents were obtained in 2016; however, the batch numbers are no longer available. The ingredients listed in Table 1 may differ from the current formulations of the compounds if the manufacturers have since modified the compositions. These potential changes should be considered when using our results as a reference in subsequent research. Additionally, upcoming studies should examine the interaction of solely experimental solutions with the dentin surface in situ.

The present study raised many questions in need of further investigation. The deposition of different structures was observed on the dentin surface even 6 hours after intraoral exposure. Since the occlusion of dentinal tubules may be lost after tooth brushing or an acidic diet, it is necessary to investigate the mechanical and chemical resistance of desensitizing agents. Furthermore, the role of the pellicle remains unclear, as no test was performed without intraoral exposure. It is also uncertain how the thickness of the pellicle influences the direct interaction of these substances with the dentin surface. Despite the fact that the dental surface is always covered by a pellicle under oral conditions, any substance is able to occlude dentinal tubules under in-vitro conditions. Finally, the results of the present exploratory study can be verified with a sufficient number of subjects and additional analyses on the permeability of occluded dentinal tubules, taking into account the bovine origin of specimens, as bovine dentin has a larger number of tubules than human dentin.^51^

In summary, the present study uncovered several novel findings related to treating dentin hypersensitivity using desensitizing agents. Notably, these agents led to the deposition of vesicular and crystalline structures on the dentin surface and within the pellicle, even after 6 hours of intraoral exposure, contributing to the occlusion of dentinal tubule orifices. This study considers the role of the pellicle in these processes, using high-resolution TEM to visualise both organic and inorganic deposits. The presence of a pellicle was found to influence the deposition and occlusion processes, highlighting the need for further investigation into the mechanical and chemical resistance of desensitizing agents under in-situ conditions.

## Conclusions

Taking into account the limitations of the exploratory study design, the present study provides new insights into the interaction of common desensitisers with the dentin surface and how different factors and structures are involved in the occlusion of dentinal tubules. Under in situ conditions, the dentinal tubules are occluded by a combination of organic and inorganic structures, whereby the desensitizing agents interact not only with the dentin surface but also with salivary proteins and the pellicle. Of all the test substances, the experimental solutions containing hydroxyapatite nanoparticles resulted in substantial deposition of inorganic structures within the tubules.

## Conflict of interest

None disclosed.
